# The Role of S100A12 as a Systemic Marker of Inflammation

**DOI:** 10.1155/2012/907078

**Published:** 2012-07-03

**Authors:** B. Meijer, R. B. Gearry, A. S. Day

**Affiliations:** ^1^Departments of Paediatrics and Medicine, University of Otago-Christchurch, P.O. Box 4345, Christchurch 8140, New Zealand; ^2^Departments of Paediatrics and Gastroenterology, Christchurch Hospital, Christchurch 8140, New Zealand

## Abstract

S100A12 is a member of the S100 family of calcium-binding proteins with important extracellular activities. In recent years, investigators across a number of fields have delineated the patterns of S100A12 expression in a variety of conditions. These data suggest that S100A12 can be used as a valuable serum inflammatory marker.

## 1. Introduction

S100A12, also known as Calgranulin C, MRP6, or EN-RAGE (extracellular newly identified receptor for Advanced glycation endproducts binding protein), is a calcium-binding pro inflammatory protein predominantly secreted by granulocytes [1]. S100A12 was first described in humans by Guignard and colleagues in 1995 [2] and has subsequently also been identified in other mammals, including pigs, dogs and cows [3–5]. 

Over the last 15 years, there has been an increasing interest in the function of this protein and in its role as an indicator of inflammation. This paper aims to review the patterns of serum S100A12 in a diverse range of inflammatory conditions and outlines the evidence supporting this protein as an inflammatory marker. This paper does not consider the role of faecal S100A12 as an indicator of gut inflammation. 

## 2. The S100 Protein Family

S100A12 is a member of the S100 family of proteins, which, in humans, consists of at least twenty-five EF-hand (*α*-helix-loop-*α*-helix) calcium-binding proteins of low molecular weight (9–14 kDa) [6]. The name S100 is derived from the fact that these proteins are 100% soluble in ammonium sulfate at normal pH. 

In 1965, Moore was the first to identify the S100 proteins [7]. Subsequently, additional members of this family have been identified and characterised. Most of these proteins are encoded by genes within a closely defined area of 1q21 [8]. The S100 proteins have 25–65% identity at the amino acid level and all contain two EF-hand motifs separated by a linker region [8]. These linker regions and the C-terminal extension region are the most variable components amongst the family of S100 proteins. 

The EF-hand motifs are each able to bind one calcium ion. When calcium binds to this section of the protein, almost all S100 proteins undergo a conformational change resulting in a concave hydrophobic surface in the C-terminal domain. This forms a target recognition site thus enabling selective interaction with a host of specific protein or peptide targets. 

The S100 proteins are found in various forms, including homodimers, heterodimers, and tetramers, which are thought to have implications for the function of the proteins. For example, the heterodimer of S100A8 and S100A9 (known as calprotectin) has a different range of activities to the individual proteins [7]. Most of the S100 proteins are thought to have intracellular functions. However, S100A12, (along with family members S100A8 and S100A9), is also recognised to have important extracellular activities. 

## 3. S100A12

The S100A12 gene is approximately 4.1 kbp long and consists of three exons, which are divided by two introns of 900 and 400 bp. The first exon is untranslated and comprises 48 nucleotides. A classical TATA box (TATAAA) is located 30 nucleotides upstream of the transcription initiation site. The protein is encoded by sequences in exons 2 (138 nucleotides) and 3 (138 nucleotides) [1]. The amino-acid structure of S100A12 was sequenced by Ilg and colleagues in 1996 [9]. 

Gene expression of S100A12 in humans is almost completely restricted to neutrophil granulocytes. Although expression by monocytes is demonstrated, this is much less than seen in neutrophils [2]. In healthy individuals, expression of S100A12 can be found in tissue and organs where neutrophils and monocytes/macrophages are common, such as the spleen and lung. S100A12 is located predominantly in the cytosol of the granulocyte but translocates to membrane and cytoskeletal components following the interactions with calcium. When secreted extracellularly, S100A12 contributes to innate immune responses. The activities of S100A12 include chemotactic activity and activation of intracellular signalling cascades leading to cytokine production and induction of oxidative stress [10, 11]. 

## 4. RAGE and sRAGE

The best known target protein of S100A12 is RAGE (receptor for advanced glycation endproducts). In addition, it is suggested that S100A12 also uses an independent receptor to affect neuronal differentiation [12]. Ligation of S100A12 with RAGE is followed by activation of intracellular signal cascades (such as MAP-kinase and nuclear factor (NF)-*κ*B) leading to production of proinflammatory cytokines (including tumor necrosis factor (TNF)-*α* and Interleukin (IL)-1*β*) and increased expression of adhesion molecules (such as intercellular adhesion molecule (ICAM)-1 and vascular cell adhesion molecule (VCAM)-1). The net effect of these responses is to mediate pro-inflammatory effects on lymphocytes, endothelial cells, neutrophils, and mononuclear phagocytes [10]. 

Soluble RAGE (sRAGE) corresponds to the extracellular domain of RAGE lacking cytosolic and transmembrane domains. As the N-terminal “V” type domain is included, sRAGE has the same ligand-binding specificity as RAGE. Consequently, sRAGE can act as a “decoy” by binding S100A12 (or other pro inflammatory ligands with a binding affinity to RAGE) thereby preventing ligation with membrane RAGE. Levels of sRAGE are reduced in various types of auto-immune diseases, such as systemic lupus erythematosus (SLE), juvenile idiopathic arthritis (JIA, systemic-onset), and Kawasaki's Disease [13–15]. In contrast, sRAGE was elevated in one study of patients with Inflammatory Bowel Disease [11]. In some settings sRAGE may be a useful biomarker itself; for instance a lower level of sRAGE appears to be associated with a two- to three fold higher risk of the development of pancreatic cancer [16].

## 5. Serum S100A12 as a Biomarker

S100A12 appears to be of clinical value in the evaluation and assessment of a number of inflammatory disorders. Recent data demonstrating the importance of S100A12 in various conditions will be reviewed in the following sections. 

### 5.1. Acute Otitis Media

Acute otitis media (AOM) is a condition commonly seen in children and may be complicated by hearing loss and meningitis [17]. A recent study examined the pattern of serum S100A12 in a group of 116 young children, with measurement of concentrations before, during, and following an episode of AOM [18]. Serum S100A12 concentrations rose significantly from baseline to a peak level at the onset of AOM. In addition, the elevated concentrations returned to normal following resolution of AOM. Intriguingly, the S100A12 response was evident only in AOM consequent to infection with two particular bacterial causes (*Streptococcus pneumoniae* and *Haemophilus influenzae*) and not with other bacterial or viral causes.

Although this study did not include an age-related control group, the changes consequent to acute bacterial infection suggest that S100A12 could be of clinical value in diagnosis and management of AOM. However, additional confirmatory studies are required. 

### 5.2. Behçet's Disease

Behçet's disease (BD) is a multisystemic inflammatory disease, which causes superficial lesions (such as oral aphthoid and genital ulceration), as well as vascular, gastrointestinal, and neurologic system involvement [19]. Han et al. [19] conducted a prospective study involving a group of ten patients (mean age 38.6 ± 9.2 years, 2 male) with proven BD. Serum S100A12 concentrations were elevated in all ten individuals, regardless of the current state of disease activity. In addition, S100A12 concentrations appeared to correlate with the activity score of BD.

This study involving a small number of patients is currently the only study evaluating S100A12 in BD. Additional studies are required to more clearly define the potential clinical role(s) of S100A12 measurement in this chronic inflammatory condition. 

### 5.3. Familial Mediterranean Fever

Familial Mediterranean fever (FMF) is an auto inflammatory disorder caused by mutations within the *MEFV (Mediterranean Fever) *gene, which lead to ongoing activation of pro inflammatory pathways [20]. This condition is seen more commonly in particular ethnic groups, especially those living in the Eastern Mediterranean area, and is characterised by recurrent fever in conjunction with local and systemic inflammatory responses [21].

Two studies have reported the patterns of serum S100A12 in individuals with FMF. Kallinich et al. [20] conducted a prospective cohort study in 52 children and adolescents with FMF. This group included 28 children with stable FMF controlled with colchicine, 19 with unstable FMF (ongoing symptoms despite administration of colchicine), 7 with newly diagnosed FMF, and five asymptomatic children with known MEFV mutations. S100A12 concentrations were associated with disease state (unstable FMF (6260 ± 2120 ng/mL) versus stable FMF (440 ± 80 ng/mL), *P* < 0,001). Furthermore, the highest concentrations of serum S100A12 were seen in the newly diagnosed FMF group (33,500 ± 22,200 ng/mL). In addition, a significant increase in serum S100A12 concentrations was also seen in asymptomatic children with MEFV mutations compared to healthy controls.

A further study has also demonstrated increased production of S100A12 in patients with FMF [22]. S100A12 concentrations were found to be more than 100 fold higher in a group of 17 children (median age 11.7 years) with FMF than in a healthy control group (6720 ± 4960 ng/mL versus 50 ± 10 ng/mL; *P* < 0.001). These two studies indicate that S100A12 may be a useful marker of disease control in FMF and suggest that S100A12 may play a role in the inflammatory events seen in FMF. 

### 5.4. Kawasaki's Disease

Kawasaki's disease (KD) is an acute vasculitis affecting small- and medium-sized arteries, which can lead to long-term cardiac complications [15]. The diagnosis of KD currently relies primarily upon clinical features, along with echocardiography to detect coronary vascular changes. 

Elevations in serum S100A12 during acute KD has been demonstrated in a number of clinical studies [15, 23, 24]. In one prospective, cohort study serum S100A12 concentrations in a group of 50 patients with KD were found to be substantially higher than the levels in a group of 50 healthy controls (450 ± 106 ng/mL versus 60 ± 15 ng/mL, *P* < 0.001) [15]. The response of serum S100A12 to therapy was assessed in a different group of 31 children with active KD. [23]. In respond to gammaglobulin therapy, serum levels of S100A12 dropped quickly after the start of the treatment (from 463 ng/mL to 184 ng/mL in the first 24 hours; *P* < 0.0001). This change was more pronounced than that of CRP values over the same time period (CRP fell from 82 mg/L to 51 mg/L; *P* = 0.015). In contrast, S100A12 concentrations in the three children who did not respond to gammaglobulin increased two to three-fold over the first 24 hours. 

Similar findings were reported in another cohort of children [24]. In addition, clinical and in vitro studies indicated that expression of S100A12 in neutrophils correlated with response to gammaglobulin therapy.

The relationship between the production of S100A12 and sRAGE also appears to be important in KD. Along with the demonstration of very high levels of S100A12, Wittkowksi et al. [15] also demonstrated lower sRAGE levels in the disease group compared to the healthy controls (575 ± 97 pg/mL versus 1,298 ± 121 pg/mL, *P* < 0.001). This reversal of the balance of these two proteins would be expected to lead to a massive amount of pro-inflammatory protein available to bind to RAGE, thus contributing to and/or amplifying the inflammatory response. At present, these data indicate that S100A12 is greatly elevated in active KD, and that measurement of this protein may be helpful as an early indicator of treatment response. Furthermore, the balance between S100A12 and the decoy receptor sRAGE also appears to be important in the inflammatory responses seen in KD.

### 5.5. Glomerulonephritis

Komatsuda et al. [25] conducted a small study in a cohort of 46 patients with myeloperoxidase anti neutrophil cytoplasmic antibodies—(MPO-ANCA-) associated pauci-immune glomerulonephritis. Serum S100A12 was increased in the disease group compared to 29 healthy controls. The concentrations of serum S100A12 correlated with a number of other markers (such as peripheral white blood cell count, concentrations of serum C-reactive protein (CRP), and creatinine), but not with serum MPO-ANCA titers. In addition, serum S100A12 concentrations decreased in the following treatment in the ten treated patients. The authors conclude that S100A12 may be a useful marker of disease activity in MPO-ANCA-associated glomerulonephritis. However, further studies in other inflammatory renal diseases are awaited. 

### 5.6. Inflammatory Bowel Disease

There are a number of studies examining serum S100A12 concentrations in patients with inflammatory bowel disease (IBD). Measurement of S100A12 and S100A8/A9 in stool samples has been established as a sensitive and specific indicator of gut inflammation, [26–28]—however the role of serum S100A12 is less clear. 

Leach et al. [29] undertook a cohort study to determine serum S100A12 concentrations in 39 children with IBD (mean age 9.7 ± 4.5 years: 29 with Crohn's disease (CD), 4 with ulcerative colitis (UC), and 6 with IBD unclassified) and 33 age-matched non IBD controls. Serum S100A12 concentrations were greater in the IBD group compared to the non-IBD group (median 196 (27–14,810) ng/mL versus median 82 (15–4242) ng/mL, *P* < 0.01). Although serum S100A12 was significantly increased in the children with CD (median 239 (27–14,810) ng/mL), the median level was higher in those with UC (median 750 (247–1391) ng/mL) and not elevated in the IBD unclassified group (median 94 (40–294) ng/mL). This may reflect the small numbers of patients in the latter two groups.

Elevated serum S100A12 was also demonstrated in a study of 74 adult patients with IBD [30]. Forty of these patients were diagnosed with CD, and 34 with UC. High levels of S100A12 were seen in active CD (470 ± 125 ng/mL, *P* < 0.001) and active UC (400 ± 120 ng/mL, *P* < 0.001) compared to healthy individuals (75 ± 15 ng/mL). The subjects with inactive CD also had elevated S100A12 (215 ± 95 ng/mL, *P* < 0.05) compared to healthy subjects. In this cohort, serum S100A12 concentrations were able to distinguish between active and inactive CD (470 ± 125 ng/mL versus 215 ± 95 ng/mL, *P* < 0.01) and also able to differentiate between active and inactive UC (400 ± 120 ng/mL versus 115 ± 55 ng/mL, *P* < 0.001). 

A further study included 201 adult subjects: 64 with UC, 64 with CD and 73 with Irritable Bowel Syndrome (IBS) [6]. A normal healthy control group was not included. Serum S100A12 concentrations were higher in both IBD groups compared to the IBS group (*P* = 0.001 for both comparisons). S100A12 levels correlated with some standard markers of inflammation, particularly CRP. The authors calculated that a cut-off of 54.4 ng/mL could distinguish between IBD and IBS, but with sensitivity of 66.7% and specificity of 64.4% only. When considered separately, the cut-offs for CD or UC were similar.

In a further study, Brinar et al. [31] evaluated serum S100A12 in 300 adults with IBD (150 CD and 150 UC), 100 non-IBD inflammatory controls (including diverticulitis, infectious enterocolitis, and ischaemic colitis) and 143 healthy controls. Significantly elevated serum S100A12 concentrations were seen in both IBD groups (median 242 ng/mL for CD and median 223 ng/mL for UC) and non-IBD inflammatory controls (median 94.7 ng/mL) compared with healthy individuals (53.5 ng/mL, *P* < 0.01). S100A12 was higher in endoscopically active UC compared with inactive disease (357 ng/mL versus 121 ng/mL, *P* = 0.009). However, no correlation was found between the activity of CD and S100A12 concentrations (156 ng/mL in active disease versus 307 ng/mL in inactive disease, *P* = 0.183). The authors concluded that S100A12 could not be used as an accurate biomarker of inflammation [31]. The differences in these results could be explained by the use of different ELISA systems used in the two studies, emphasising the importance of consistent methodology. Although the overall interpretation suggests that serum S100A12 levels may be clinically useful, further studies in paediatric and adult patient cohorts are required to more clearly define these roles.

### 5.7. Juvenile Idiopathic Arthritis

Several studies have examined the patterns of serum S100A12 in juvenile idiopathic arthritis (JIA). JIA is defined as an idiopathic arthritis with onset before the age of 16 years, which persists for at least 6 weeks. JIA includes different subtypes depending on the extent and pattern of joint involvement: these are systemic-onset JIA (SoJIA), polyarticular JIA (polyJIA), enthesitis-related JIA (ERA), or oligoarticular JIA (oligoJIA) [22, 32].

Wittkowski et al. [22] enrolled 60 children with newly diagnosed SoJIA in a prospective cohort study. This group had mean age of 9.1 (range 1.8–18.1) years. Serum S100A12 concentrations in the children with SoJIA were more than 100-fold greater than those in a comparative group of healthy children (7,190 ± 2,690 ng/mL versus 50 ± 10 ng/mL, *P* < 0.001). In addition, S100A12 levels were able to differentiate SoJIA from infectious states with sensitivity of 66% and specificity of 94%, unlike CRP or ESR measurements.

Similar findings were demonstrated in a study of 20 Egyptian children with long-standing JIA (mean disease duration of 5 years) [33]. Serum S100A12 concentrations were around 8-fold higher in the disease group than the control group overall, with higher concentrations seen in the patients with SoJIA compared to other subtypes. Furthermore, S100A12 correlated closely with clinical, biochemical, and radiological markers of disease severity. This study did not detail current therapies, which may have influenced the patterns observed in these children.

In a more recent study, Myles et al. [32] evaluated serum S100A12 in 121 Indian children with JIA (101 ERA, 10 polyJIA, and 10 SoJIA) along with 45 healthy controls. Serum sRAGE was also measured in these children. Median S100A12 concentrations were significantly higher (*P* < 0.01), and sRAGE concentrations were significantly lower (*P* < 0.0001) in all subgroups of JIA compared to healthy controls. In addition, S100A12 concentrations were similar across the three groups of JIA.

Overall, these three studies suggest that S100A12 measurement provides a useful and clinically relevant assessment of inflammation in these disease groups. The value of S100A12 in assessing response to therapy or predicting longer term outcomes remain unclear.

### 5.8. Rheumatoid and Psoriatic Arthritis

One study has examined the patterns of serum S100A12 in adult patients with these types of arthritis. Foell et al. [34] included 22 patients with Psoriatic Arthritis (PsA), 9 patients with Rheumatoid Arthritis (RA), and 11 with Seronegative Arthritis (SA). These groups were compared to 30 healthy adult controls. Significant (*P* < 0.05) increases in serum S100A12 concentrations were seen in each of the arthritis groups compared to healthy controls (60 ± 20 ng/mL), with the highest concentrations seen in RA patients (340 ± 90 ng/mL) [34]. There was a moderate elevation in PsA (260 ± 60 ng/mL) and a smaller but still significant elevation in SA (190 ± 20 ng/mL). 

### 5.9. Cystic Fibrosis

Cystic fibrosis (CF) is a systemic disorder in which lung injury due to chronic airway inflammation is the primary cause of morbidity and mortality [35]. Complications are caused by bacterial infections, especially *Staphylococcus aureus* and *Pseudomonas aeruginosa.* These bacterial infections cause a neutrophilic inflammation, which contributes to progressive tissue damage. This is the main reason for hospital admission and antibiotic treatment. 

Foell et al. [35] measured serum S100A12 in 35 patients with CF (18 inpatients with an acute exacerbation and 17 outpatients without exacerbations) and compared these results to healthy volunteers. Serum S100A12 concentrations were elevated in the CF patients with acute exacerbations compared to healthy controls (median 225 ng/mL versus 46 ng/mL, *P* < 0.001), and these values fell (76 ng/mL: *P* < 0.01) after two weeks treatment with intravenous antibiotics. In addition, serum S100A12 was also significantly elevated in CF outpatients without exacerbations compared to the controls (105 ng/mL, *P* < 0.01), suggestive of ongoing airways inflammation.

### 5.10. Other Respiratory Disorders

Data shows that S100A12 is elevated in bronchoalveolar fluid of patients with asthma and respiratory distress syndrome (RDS) [10, 36]. RDS is primarily seen in premature infants and is the consequence of incomplete pulmonary maturation [37]. To date, just one study has examined serum S100A12 in the context of RDS [38]. Loughran-Fowlds et al. [38] measured serum S100A12 in a cohort of premature infants and demonstrated lower serum S100A12 on day 1 of life in infants who developed RDS (*P* < 0.01).

More studies examining the serum values of S100A12 in respiratory disorders are required before a conclusion can be made about the value of serum S100A12 in these disorders.

## 6. Renal Disease and Atherosclerosis

The relationship between sRAGE and S100A12 also has been studied in patients with end-stage renal disease requiring peritoneal dialysis (PD) [39]. Patients with PD had significant elevation of sRAGE (1329.6 versus 565.8 pg/mL; *P* < 0.01) and S100A12 (143.3 versus 64.4 ng/mL; *P* < 0.01) compared with healthy controls. CRP, fibrinogen, and IL-6 were also markedly increased in the PD group (*P* < 0.001). Furthermore, a correlation was found between serum S100A12 levels and the vascular calcification score (VCS; *r* = 0.315, *P* = 0.015) and carotid intima-media thickness (cIMT; *r* = 0.391, *P* = 0.002). In contrast, sRAGE was negatively correlated with VCS (*r* = −0.248, *P* = 0.020) and cIMT (*r* = −0.375, *P* < 0.001). These findings support important relationships between high serum S100A12 levels and the progression of atherosclerotic vascular complications consequent to the induction of systemic inflammation. On the other hand, sRAGE may have an opposite effect, as this protein acts as a “decoy” for S100A12, providing an anti-inflammatory, anti-atherogenic, and anti vascular calcification effect, particularly in diabetic patients.

## 7. Measurement of Serum S100A12

If assessment of S100A12 is to be considered outside the research context (and for routine clinical practice), it will be essential to ensure consistent approaches are available for collection and assay of samples. Collection of serum, rather than plasma, is important, with gel-containing tubes recommended [40]. Calcium and heparin exposure also influence measurement of S100A12 levels [40]. 

Although earlier studies utilised in house immunoassays, commercial enzyme-linked immunoassays (ELISAs) are now available. Variation and inconsistency between assay kits is a potential confounding factor [31, 41]. Standardised methodology and reliable consistent ELISA kits will be important in considering further roles for the measurement of this protein.

## 8. Summary and Conclusions

S100A12 is a small protein expressed primarily by neutrophils with a number of important extracellular activities relevant to innate and acquired inflammatory responses. Recent studies have shown elevated serum levels of S100A12 in a number of acute and chronic inflammatory conditions. 

The current literature shows that serum S100A12 can be used as marker of disease activity across a range of inflammatory states. Although serum S100A12 appears to have a particular role in KD as a disease-specific marker (as there are no current specific biochemical tests for this condition), it also has roles in assessing the early response to therapy in KD. Measurement of S100A12 may also have a role in the assessment of response in AOM and CF. 

Further clinical studies are now required to fully elucidate the clinical relevance of S100A12 measurement across these various conditions. Such studies will establish if this protein has relevance beyond that of an inflammatory mediator.
